# Coronary Involvement in Behçet's Disease: what are its Risks and Prognosis? (Rare Cases and Literature Review)

**DOI:** 10.21470/1678-9741-2019-0003

**Published:** 2019

**Authors:** Unsal Vural, Mehmet Kizilay, Ahmet Arif Aglar

**Affiliations:** 1 Doktor Siyami Ersek Gogus Kalp ve Damar Cerrahisi Egitim ve Arastirma Hastanesi - Cardiovascular Surgery, Istanbul, Turkey.

**Keywords:** Behcet Syndrome, Coronary Artery Diseases, Aneurysm, False, Thrombosis, Coronary Artery Bypass

## Abstract

**Objective:**

In our clinic, we aimed to investigate the effect of preoperative risk factors and postoperative complications on reoperation and mortality in cases with Behçet's disease which presents very rare coronary artery involvement.

**Methods:**

Thirteen patients with Behçet's Disease who had undergone coronary artery bypass grafting in our center between 2003 and 2015 were analyzed. We evaluated the clinical and laboratory findings, complications and mortality rates of our patients in light of the literature.

**Results:**

The mean age was 38.5 (30-55; 3 women). The mean time from onset of Behçet's disease to coronary artery disease was 4,7 (3-11) years. Fifty-four percent of the patients were asymptomatic. Coronary artery disease of these was exposed while peripheral vascular surgery was planned due to complications of Behçet's disease. Symptomatic patients presented angina pectoris (31%), acute coronary syndrome (8%) and arrhythmia (8%). In coronary pathology of patients, distal type obstruction (31%), aneurysm and pseudoaneurysm (31%), proximal segment thrombus (15%), chronic type stenosis and occlusions (31%) were present. Early mortality (15%) was due to acute myocardial infarction while the late mortality (15%) was due to cerebral and gastrointestinal bleeding. Reoperation was due to bleeding in one case on the 1st postoperative day and due to acute pulmonary embolism in another case in the 3^rd^postoperative year.

**Conclusion:**

In Behçet's disease, coronary artery bypass grafting is a procedure with high mortality, especially in the acute period. The on-pump surgery technique in these cases can be safely performed for multiple bypasses and in patients above 40 years old.

**Table:** 

Abbreviations, acronyms & symbols	
BD	= Behçet's disease
CABG	= Coronary artery bypass grafting
CRP	= C-reactive protein
CT	= Computerized tomography
ESR	= Erythrocyte sedimentation rate
GIS	= Gastrointestinal system
INR	= International normalized ratio
LAD	= Left anterior descending artery
LVEF	= Left ventricular ejection fraction
LIMA	= Left internal mammary artery
RCA	= Right coronary artery
RIMA	= Right internal mammary artery

## INTRODUCTION

Behçet's disease (BD) was first described in 1937. Data for Behçet's disease differ due to the fact that the disease is detected at different rates in different geographical regions and ethnic groups and is rare. It is mostly seen in the Eastern Mediterranean and the Far East (along with the Silk Road). The incidence in Turkey is 0.3% whereas it is 0.02% in Japan, Korea, Iran, Iraq, and Saudi Arabia ^[1]^. It is a chronic inflammatory disease that is more severe among young men and exhibits prolonged remission periods and relapses of decreasing severity as the age increases. The onset is severe and progression is fast in 30 to 40-year-old males (male/female ratio 3:1). Mortality varies according to ethnicity, geographic and genetic characteristics^[1]^.

In its pathogenesis, it is thought to be an autoinflammatory disease rather than an autoimmune disease due to the increased response of innate or acquired immune systems to environmental antigens and autoantigens. In addition, hypergammaglobulinemia and female dominance seen in classical autoimmune diseases are not seen in BD. Indeed, BD is a ‘neutrophilic vasculitis/perivasculitis'. Generally, vascular manifestations occur in association with signs of inflammatory activation (such as fever and constitutional symptoms)^[2]^. The presence of anti-lymphocyte and anti-cardiolipin antibodies is shown in BD and is used in the diagnosis of the disease. The prevalence of BD in some ethnic groups supports the role of genetic mechanisms in pathogenesis. The most discussed genetic marker is HLA-B51, a subclass of the HLA class^[2]^. Although the incidence of BD is higher in family members, the disease-related complications are lower in those ^[3]^.

Vascular involvement in BD is reported between 8-39% in different series and can involve both arteries and veins of any diameter. Arterial involvement in BD is far less common than venous involvement (20% *vs*. 80%). The frequently involved major arteries are the abdominal and thoracic aorta and pulmonary, iliac, and femoral arteries. However, coronary arteries are rarely affected and are only reported as case presentations in the literature^[4]^. Vasculitis may manifest as an aneurysm, thrombosis or occlusion. Abdominal aorta (60%) is the most common aneurysm region in Behçet's disease, whereas ulnar, celiac, subclavian, tibioperoneal, iliac and superior mesenteric arteries have been reported as rare aneurysm regions^[5]^. Since it is mostly in the form of a saccular aneurysm, symptoms due to compression to neighboring structures are frequent and the probability of rupture is very high. Although surgery is recommended in these saccular aneurysms, it is not recommended in the acute period of inflammation^[6]^. Surgery can either be performed after the administration of immunosuppressive therapy that normalizes the acute phase reactants or in case of rupture and bleeding risk. Pseudoaneurysm formation may occur in the anastomosis line after bypass grafting, or in the percutaneous coronary intervention access site and in the stented segment of the coronary artery^[6]^.

In the light of literature, in our study, we aimed to investigate the effect of preoperative risk factors and postoperative complications on reoperation and mortality in cases with BD, which presents very rare coronary artery involvement.

## METHODS

Between 2003 and 2015, a total of 13 patients who underwent coronary artery bypass surgery at our hospital, 10 (3 female, 23%, Table 1) were diagnosed preoperatively with BD and followed up by dermatology or ophthalmology clinics and 3 (23%) of them were diagnosed postoperatively with BD, were retrospectively analyzed. The study was approved by the Health Sciences University Haydarpaşa Numune Hospital Ethics Committee (Number 28001928-508.05). Informed consent was waived by the Institutional Review Board because of retrospective nature of the study. Anamnesis and clinical examination findings, coronary angiography, and whole-body CT angiography were used for the diagnosis. Clinical (additional vascular pathology, additional symptoms of BD) and laboratory findings (CRP, ESR, leukocyte and lymphocyte counts), as well as complications seen during and after the operation, and mortality rates were analyzed in the light of the literature (Tables 1 and 2).

**Table 1:** Preoperative demographic characteristics of patients and additional clinical and laboratory findings of Behçet's disease.

		Male (n=10)		Female (n=3)	
		Mean	SD	Mean	SD
Age		40.4	7.3	32	2.6
LVEF		36.5	82	45	13.2
ESR		39.9	17.4	26	8.7
Leukocyte		11,985	4,376	9,133	2,579
		**n**	**%**	**n**	**%**
Myocardial infarction		3	30	0	0
Angina pectoris		6	60	1	33
Oral aphthosis		8	80	3	100
Genital aphthosis		8	80	3	100
Uveitis		8	80	3	100
Retinal vasculitis		3	30	1	33
Cataract		3	30	0	0
Pericarditis		2	20	0	0
Joint manifestations	Arthralgia	2	20	1	33
	Monoarthritis	1	10	0	0
Skin manifestations	Erythema nodosum	2	20	0	0
	Pseudofolliculitis	5	50	3	100
Gastrointestinal system	Peptic ulcers	1	10	0	0
	Gastroduodenitis	2	20	0	0
Neurological events	Central	1	10	1	33
	Peripheral	1	10	0	0
Hepatosplenomegaly		2	20	0	0
HLA-B51		7	70	1	33
Pathergy test		7	70	2	67

ESR=erythrocyte sedimentation rate; LVEF=left ventricular ejection fraction

**Table 2:** Distribution of extracardiac vascular involvement, number of distal anastomoses and complications.

		n=13	%
Extracardiac vascular events	Abdominal aneurysm	2	15
	Deep vein thrombosis	3	23
	Femoral pseudoaneurysm	2	15
	Aortic thrombosis	1	8
	Radial pseudoaneurysm	1	8
	Popliteal aneurysm	1	8
	Pulmonary thrombosis	1	8
CABG	CABGx1	0	0
	CABGx2	4	31
	CABGx3	5	38
	CABGx4	4	31
Mortality	Early	2	15
	Late	2	15
Reoperation	Early	1	8
	late	2	15

CABG=coronary artery bypass grafting

### Surgical Method

**Fig. 1:**
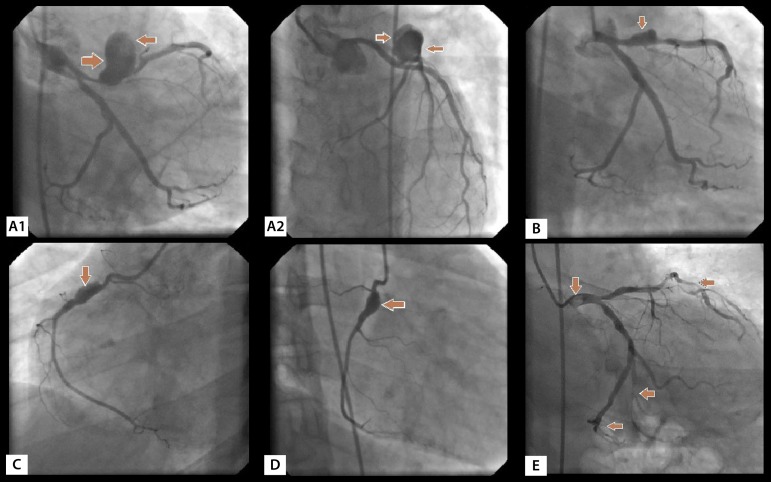
In BD: A1) Right anterolateral appearance of pseudoaneurysm formation in left anterior descending artery (LAD) proximal (34-year-old male); A2) Anteroapical view of pseudoaneurysm in LAD. B) Aneurysmal dilatation in proximal LAD (right anterolateral position); C) Aneurysmal dilatation in proximal RCA (left anterolateral position, 36-year-old male); D) Right anterolateral position of the aneurysm in proximal RCA; E) Thromboembolism and distal embolization in the left main coronary artery (45-year-old male).

The operations were performed under general anesthesia with median sternotomy. Myocardial protection was achieved with antegrade cold blood cardioplegia delivered in every 20 minutes and moderate hypothermia (28-32°C). Left ventricular decompression was achieved through the right superior pulmonary vein. After the completion of distal coronary anastomoses and proximal anastomoses in the aorta, the cross-clamp was removed. The patient was decannulated after heating. Primary repair of pseudoaneurysm was performed in the same session in two patients with femoral pseudoaneurysm. In one patient, a mobile thrombus originating from the non-coronary sinus of the aorta was removed by aortotomy during the bypass. Radial pseudoaneurysm formed postoperatively was repaired in a separate session. Abdominal saccular aneurysms in two patients were successfully repaired by endovascular stent grafting before coronary surgery. In one patient, suprapopliteal aneurysm was repaired by saphenous vein interposition in the femoropopliteal region with a separate operation. In patients with coronary aneurysm, the aneurysmal segment was ligated and bypassed (Figure 1).

Medical treatment was continued for 2-8 weeks for acute phase reactants (CRP and lymphocyte count, sedimentation rate) to decrease in non-emergency cases. Patients who did not have a diagnosis of BD preoperatively but happened to be diagnosed with BD postoperatively, and who were known to have BD preoperatively but presented urgency (total of 5 cases, 38.5%) undergone operation in the acute phase of the disease. Postoperatively, colchicine was continued in all cases. Corticosteroids (1-2 mg/kg/day) and immunosuppressive therapy (azathioprine, cyclosporine A, and cyclophosphamide) were initiated on the 7^th^ postoperative day since it was thought that the risk of infectious complications were reduced. From the 1^st^postoperative day, antiaggregant therapy was used against the risk of arterial and venous thromboembolism. Local treatment was used for oral and genital ulcers. Since the follow-up and treatment of patients with BD required a multidisciplinary approach, the cardiologist, cardiovascular surgeon, dermatologist, rheumatologist, ophthalmologist, and neurologist were involved in the treatment process. In the postoperative period, the patient was cautioned to be followed up by the rheumatologist and dermatologist of the center where the primary treatment of the disease was carried out. Generally, there was no consensus on the duration of immunosuppressive therapy, but it was continued for at least two years and treatment was stopped in patients with remission for at least two years. However, in patients with vasculitis and uveitis, longer periods of treatment were needed to achieve complete remission.

### Statistical Analysis

Continuous variables were defined by mean and standard deviation while categorical variables were defined by numbers and percentage (%). Spearman correlation analysis was used to evaluate the relationship between age and complications. The relationship between the complications of BD and survival was analyzed by Kaplan-Meier (log-rank) test. For the analyzes, the Statistical Package for Social Sciences Statistical Software version 18.0 (SPSS Inc., Chicago) was used. *P*-value was considered significant in *P*<0.05.

## RESULTS

**Fig. 2:**
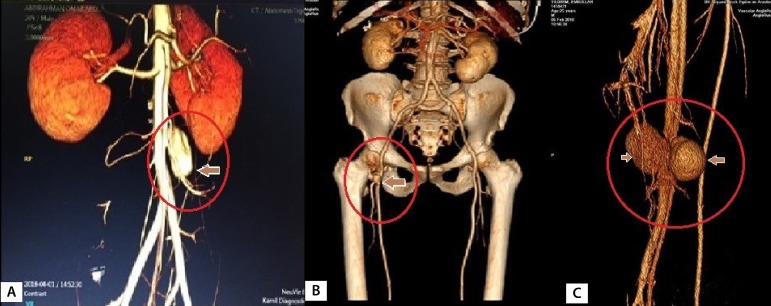
A) Fistulization of supra-popliteal arterial pseudoaneurysm to the popliteal vein in a patient with BD and history of coronary artery bypass (35-year-old female patient with no history of trauma); B) CT angiographic appearance of pseudoaneurysm formation at the right common femoral artery access site (39-year-old male patient); C) In a case with BD, the infrarenal saccular aneurysm in the abdominal aorta (55-year-old male patient; the lesion was stented before CABG).

**Fig. 3:**
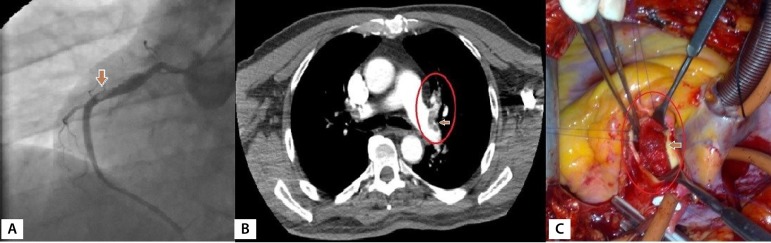
A) Left anterolateral view of the thrombus in the proximal RCA (30-year-old female patient); B) After CABG, the patient with BD developed pulmonary embolism following deep vein thrombosis in the third postoperative year. The view of the emboli in the left pulmonary artery (55-year-old male); C) Perioperative image of mobile thrombus originating from the noncoronary sinus of Valsalva (30-year-old female patient, the aortic valve was structurally and functionally normal).

The mean age was 38,5 (30-55, 3 out of 13 were women). The mean time from onset of BD to coronary artery disease was 4.7 (3-11) years. Fifty-four percent (7 cases) of the patients were asymptomatic. Coronary artery disease in asymptomatic patients was detected while peripheral vascular surgery was planned due to peripheral vascular complications of BD. Symptomatic cases presented angina pectoris (31%, 4 cases), acute coronary syndrome (8%,1 case) and arrhythmia (8%, 1 case). In the coronary arteries of the cases, distal type occlusion was detected in 31% (4 cases), aneurysm and pseudoaneurysm in 31% (4 cases), thrombus in the proximal segment of the coronary in 15% (2 cases), and chronic stenosis and occlusion in 31% (4 cases). In the context of known risk factors for coronary artery disease, smoking was seen in 5 cases, hypertension in 3 cases, diabetes mellitus in 2 cases, obesity in 2 cases and hypercholesterolemia in 5 cases. Such cases included extracardiac vascular involvements, such as abdominal aortic aneurysm (Figure 2A) in 2 cases (15%), pseudoaneurysm (Figure 2B) in femoral access site in 2 cases (15%), mural thrombus in ascending aorta (Figure 3) in 1 case (8%), supra-popliteal arterial pseudoaneurysm (Figure 2C) in 1 case (8%), superficial thrombophlebitis in 3 cases (23%) and pseudoaneurysm in the radial artery access site in 1 case (8%) were detected (Table 2). Other findings related to BD are shown in Table 1. Femoral pseudoaneurysms were the result of manipulations during catheterization and radial pseudoaneurysm was a consequence of invasive arterial blood pressure monitoring. Mobile thrombus originating from the non-coronary sinus in the ascending aorta of an emergency case was removed by thrombectomy when performing coronary artery bypass.

Reoperation was required in one case due to hemorrhage on the 1^st^ postoperative day and in another case due to acute pulmonary embolism in the 3^rd^postoperative year. Pulmonary thrombectomy was performed in this case. All patients discharged received a prescription for acetylsalicylic acid, metoprolol and ramipril tablets. The cases were followed up for 5 years. At follow-up, the cases were called for control examination at each month during the first 6 months, and at every 6 months thereafter. In the first follow-up visit, CT angiography was performed for possible pseudoaneurysm formation in the anastomosis lines and this was repeated every year in cases with active disease.

**Fig. 4:**
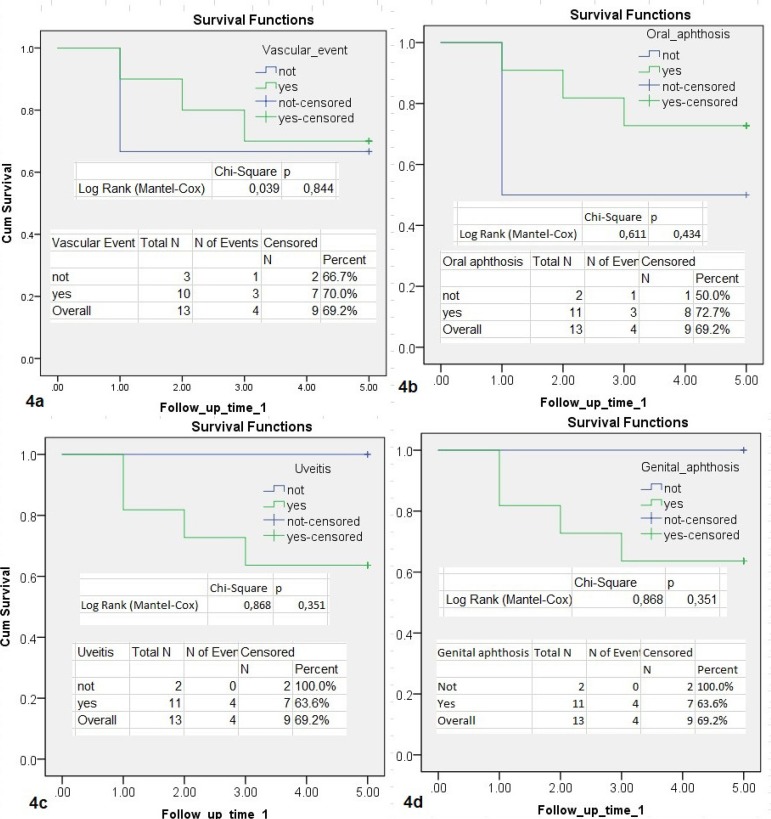
A graphical view of a 5-year survival analysis of cases according to the additional vascular event (4a), oral aphthosis (4b), uveitis (4c) and genital aphthosis (4d). It was analyzed by Kaplan-Meier (log-rank) test. There was no significant difference in mortality in terms of additional complications of the disease. One-year life expectancy is 84.6%, 5-year life expectancy is 69.2%.

**Fig. 5:**
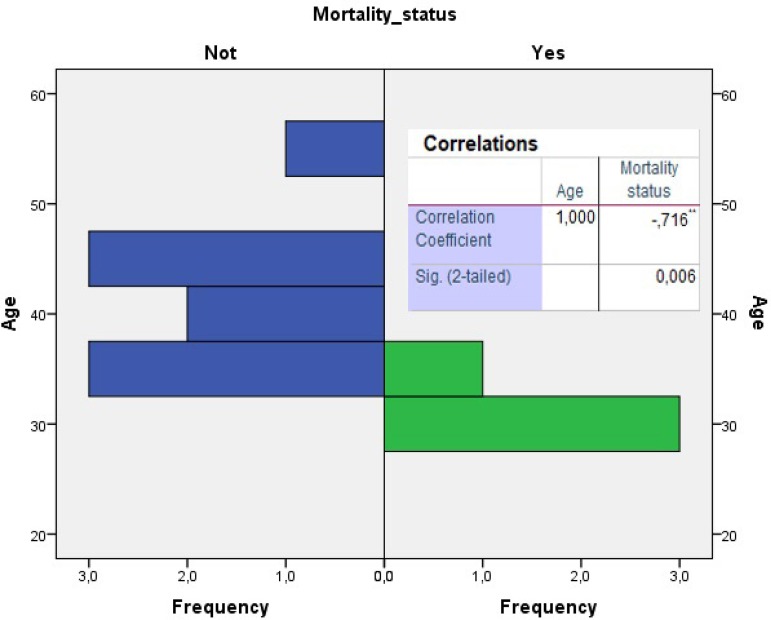
Correlation between age and mortality (significant negative correlation of 71.6%; P=0.006).

Mortalities observed in the early period (2 cases, 15%) were due to acute myocardial infarction, whereas mortalities in the late period (1-5 years) were caused by cerebral and gastrointestinal bleeding. One of these cases had undergone emergency coronary bypass. Five-year mortality rates and age distribution are also seen in Figures 4 and 5. There was a significant negative correlation between mortality and age (*P*=0.006; r= -0.716, Figure 5). In other words, mortality in younger patients was higher than in those with advanced age. All 4 deaths detected in the early and late period were male. There was no significant relationship between extracardiac vascular involvement and other findings of BD (oral aphtosis *P*=0.338; genital aphthosis *P*=0.638; uveitis *P*=0.338; joint manifestations *P*=0.364; skin manifestations *P*=0.979; gastrointestinal system *P*=0.927; HLA-B51 *P*=0.889). None of our deaths were in the acute phase of the disease.

## DISCUSSION

Arterial involvement ranges from 6.3-15.3%, according to epidemiological and clinical studies in Japan^[7]^. The main effect of the disease is in the venous system (70%)^[7]^. Arteries in BD are affected by the perivascular and endovascular inflammatory process, resulting in a unique spectrum of stenosis, thrombotic obstruction, bleeding and aneurysms (20% in an aneurysm, 19% in pulmonary embolism)^[8,7]^. Cardiac involvement manifests mostly as coronary arteritis, myocardial infarction, and aortic insufficiency. Among patients with BD, coronary artery disease is very rare and its prevalence is reported to be 0.5% on the Silk Route^[8-10]^. Ten (77%) of our cases were previously diagnosed with BD. In the other 3 (23%) cases, the diagnosis of Behçet was made with the detection of oral and genital ulcers as well as uveitis, immediately after coronary artery bypass. Arterial reconstruction in patients with BD was not recommended until near past. The causes are (I) the occurrence of additional obstructions and aneurysms beyond targeted vessel occlusion with the progression of the inflammatory disease, and (II) pseudoaneurysms occurring in the suture lines^[10]^. Although not proven, it is believed that accelerated atherosclerosis may occur due to the widespread use of steroids in this disease^[11]^.

Some pathologists, who believe that the underlying cause of systemic involvement is vasculitis, think that vasculopathy is a result of perivascular infiltration in tissues by some cytokines and T-lymphocyte mediators. Superoxide anion radicals and lysosomal enzymes released from activated neutrophils cause destructive effects. This leads to lymphocytic infiltration, vasa vasorum occlusion and transmural necrosis in the walls of the large muscular arteries. Subsequently, it causes destruction of the vessel wall, leading to deterioration of local blood flow. Continuous degeneration of the vessel wall results in a true aneurysm. In the meantime, if there is a perforation in the vessel wall, it results in pseudoaneurysm ^[12]^. Due to similar mechanisms, the suturability of vascular structures in BD is also quite low. Therefore, in some articles, it is recommended to use right internal mammary artery, left internal mammary artery and gastroepiploic artery with the off-pump technique to avoid cannulation of the ascending aorta and proximal anastomoses^[12]^.

In BD, increased thromboembolism is triggered due to endothelial dysfunction, von Willebrand factor release, platelet activation, increased thrombin and fibrin release, and antithrombin deficiency^[13,14]^. On the other hand, fibrous intimal thickening may result in acute myocardial infarction despite angiographically normal coronary arteries. It is believed that such impaired microvascular function is the cause of coronary events in these patients^[14,15]^. Coronary artery thrombi were detected in 15% of our cases. In addition, 77% of our cases had additional vascular pathologies in different areas of the body (Table 2). We started anticoagulant treatment in patients with coronary thrombosis and additional vascular thrombosis in the postoperative period. However, many researchers suggest that anticoagulant therapy should be avoided^[15,16]^. Researchers from different perspectives believe that although the formation of new aneurysms due to anticoagulants is not reported, they can activate aneurysms, especially in major organs^[14]^. The development of pulmonary embolism in one of our cases in the 3^rd^postoperative year was a result of irregular use of anticoagulant therapy, verified by unstable INR values. This case had a history of deep venous thrombosis. Although we did not detect any cerebral aneurysms in our study, neurological events were detected in 23% of our cases, regardless of the operation. We believe that this condition is the result of inflammation and, thus, occlusion of the microvascular circulation causing damage to peripheral and central nerves (neuro-Behçet's disease).

While ocular involvement, which manifests early in the disease, is the most important morbidity, vascular involvement is the most important cause of mortality. Overall mortality in BD cohort was 0.9% per year in the median follow-up in Japan. This rate is 9.8% per year in Turkey. Male gender and arterial involvement were associated with mortality in BD^[17]^. Vascular involvement is responsible for 43% of deaths in BD^[12]^. Major causes of death are major vascular disease (mainly arterial aneurysms and Budd-Chiari syndrome, 43.9%), cancer and malignant hemopathy (14.6%), and central nervous system involvement and sepsis (12.2%)^[17]^. Our early mortality rate was high (15%) due to emergent cases operated in the active phase of BD, and patients with low ejection fraction status. However, we did not have the opportunity to compare our results since there is not a larger series of patients with coronary involvement of BD in the literature. In our 5-year follow-up, our annual mortality rate (6% per year) was similar to that in the literature. Cerebral bleeding (7.7%) and gastointestinal bleeding (7.7%) were equally responsible for late mortality.

In studies comparing the incidence of ischemic heart disease in the general population and BD, rates were higher in BD (11% *vs*. 8%)^[12]^. In a study that reported a 9.1% arterial involvement rate (in 553 cases), ratios distribution of lesions such as aneurysm, occlusion, stenosis, and pulmonary vasculitis were 0.5% (n=28), 0.2% (n=11), 0.01% (n=2), 0.2% (n=11), respectively ^[18]^. In the same series, the cardiac involvement rate was 0.6% (n=37)^[18]^. Distribution of coronary lesions in our cases was as follows: 31% of distal type obstruction, 31% of proximal aneurysm and pseudoaneurysm, 15% of thrombus in the proximal segment, and 31% of chronic stenosis and occlusions. In addition to coronary involvement, venous thromboembolism in 23%, aortic aneurysm in 15%, peripheral pseudoaneurysm in 31% and aortic thrombus in 7.% of the cases were detected. In the late period, pulmonary artery emboli were detected in 7.7% of the cases. In patients with BD, some of the coronary aneurysms are seen as asymptomatic while in some cases as acute coronary syndrome due to distal embolization^[10,12,14]^. In our cases, 15.4% of the coronary aneurysms were true aneurysms while the 7.7% were pseudoaneurysms. Saccular aneurysms were also present in the abdominal aorta of patients with coronary aneurysms. Behçet disease, by especially targeting the aortic root, may cause aortitis, and thus aneurysm of the sinus of Valsalva, resulting in acute or chronic aortic valve insufficiency. In one of our cases, mobile thrombus located in the noncoronary sinus of Valsalva was detected during coronary bypass surgery (Figure 3C). The aortic valve was intact in this case. A free-floating thrombus without aneurysm in the ascending aorta indicates an underlying prothrombotic etiology^[2]^. If the thrombus appears to be relatively immobile, oral anticoagulants and antiplatelet agents, corticosteroids and immunosuppressive agents may be used. However, mobile thrombi should be treated with surgical methods. In medical treatment, BD cases' susceptibility to bleeding should also be taken into account. We used anticoagulant therapy in our cases with a history of thromboembolism. However, in one case, pulmonary embolism developed in the 3^rd^postoperative year. In addition, one of our cases died due to gastrointestinal bleeding and another one due to intracranial hemorrhage. In late mortality, we believe that the intracranial and gastrointestinal damage of ongoing inflammation aggravated by antiaggregant therapy may play a role. On the other hand, considering the case that there was interference due to pulmonary embolism, prothrombotic state should be prevented.

Technological developments allow the venous system to be screened by radionuclide venography and the arterial system by CT angiography (Figures 2A, B and C). If conventional angiography is preferred, it is necessary to be careful with its complications. Venous puncture, intravenous infusion, rapid injection, bolus infusions of contrast agent, and venous catheter placement may trigger venous thrombosis in BD. Similarly, invasive arterial procedures may cause pseudoaneurysm formation (Figure 2B). For this reason, noninvasive methods, such as Doppler ultrasonography, computerized tomography (CT) and magnetic resonance angiography, should be preferred for diagnosis. At the femoral artery access site for angiography in two cases, and at the radial artery access site for arterial monitoring in one case, pseudoaneurysms were detected. All pseudoaneurysms were repaired primarily.

The demonstration of inflammatory markers in BD (CRP, leukogram, sedimentation) is important in determining the timing of interventions for disease complications. Primary medical therapy is immunosuppressive therapy. In case of specific organ complications, different palliative treatments should be added to this treatment. However, there are also meta-analyses reporting that the administration of corticosteroids following acute myocardial infarction has been shown to slow the ventricular healing process and to prevent the scar formation and increase the risk of myocardial rupture^[19]^. Although percutaneous coronary intervention has been suggested instead of surgery in the active period of vasculitis, there are no long-term results on its complications and outcomes^[2]^. There are also articles defending the preference of the off-pump technique to prevent manipulation of the aorta^[20]^. However, the treatment strategy of coronary artery disease is not always easy due to the scarcity of vascular BD cases and the lack of large controlled studies. We performed our operations with the on-pump technique. The reasons for this were (1) collateral insufficiency due to young cases, (2) multiple distal anastomoses, (3) susceptibility to complications such as tearing in the vascular wall weakened by inflammation, and (4) vessel diameter smaller than 2 mm. In only two of our cases (15%), during the surgery, we detected a bleeding complication with the separation of the aortic cannulation site as a button. The bleeding focus was repaired with pledget sutures without any complication. In follow-up visits, all vascular structures were screened for both possible pseudoaneurysms in the anastomosis line, and the formation of new aneurysms. No new aneurysm formation was detected.

Some measures are needed to take to improve outcomes and reduce complications of surgery in patients with BD. The first of these is to provide adequate medical treatment before and after the surgery in order to reduce the inflammatory load and to facilitate the treatment of the tissues. Second, all autogenous grafts (saphenous vein, left internal mammary artery, etc.) to be implanted should be used after screening to exclude the effect of vasculitis. In our opinion, the use of any artery or vein that has not developed proliferation and aneurysmatic dilatation in the intima and media layers in cases with low acute phase reactants will have a positive effect on the results. Third, avoiding excessive manipulation of the aorta will prevent distressing bleeding. Especially in patients with an aneurysm, severe bleeding occurs due to the use of anticoagulants and thrombolytic agents. This is due to the decrease in the activation of plasminogen and fibrin in patients suffering from active disease^[14]^.

## CONCLUSION

Coronary involvement should be considered in all cases with vascular BD. Especially in young patients, we believe that the treatment strategy should be less invasive if there is no contraindication because both distal coronary artery involvement and mortality are higher in these cases. In BD, coronary artery bypass grafting is a procedure with high mortality, especially in the acute period. Therefore, in cases for whom emergency treatment is not required, we believe that surgical treatment is more appropriate after medical treatment has been completed and the acute phase has passed. In our opinion, in patients who underwent surgery in the active period of the disease, continuing the medical treatment (corticosteroids, immunosuppressants, etc.) after surgery will decrease the postoperative complication rate. The on-pump surgical technique in BD patients can be safely performed, especially for multiple bypasses and in patients above 40 years old. Although our number of cases was insufficient to give a definitive judgment, we did not find a relationship between mortality rates and the acute period of the disease and the use of anticoagulants. However, we believe that it would be more appropriate to evaluate the results of the operation with larger series and long-term follow-up.

**Table:** 

Authors' roles & responsibilities	
UV	Substantial contributions to the conception or design of the work; or the acquisition, analysis, or interpretation of data for the work; final approval of the version to be published
MK	Drafting the work or revising it critically for important intellectual content; final approval of the version to be published
AAA	Final approval of the version to be published
